# Factors Associated with Early Versus Late Uptake of the COVID-19 Vaccine during Pregnancy over Time in Australia: A Population-Based Cohort Study

**DOI:** 10.3390/vaccines11111713

**Published:** 2023-11-13

**Authors:** Michelle L. Giles, Sushena Krishnaswamy, William Coote, Mary-Ann Davey

**Affiliations:** Department of Obstetrics and Gynaecology, Monash University, Clayton, VIC 3168, Australia; sushena.krishnaswamy@monashhealth.org (S.K.); wdcoote@gmail.com (W.C.); mary-ann.davey@monash.edu (M.-A.D.)

**Keywords:** pregnancy, COVID-19, vaccination, influenza, immunisation

## Abstract

Background: Pregnant women are at an increased risk of hospitalisation, admission to the intensive care unit, mechanical ventilation, and death from SARS-CoV-2 infection. The aim of this study is to determine the predictive factors associated with COVID-19 vaccine uptake during pregnancy over time in a population with a high background uptake of maternal influenza and pertussis vaccination. Methods: This is a population-based, cohort study of all pregnant women who gave birth in Victoria, Australia between 1 July 2021 and 30 June 2022. Data from the Victorian Perinatal Data Collection were analysed using univariable and multivariable logistic regression. Results: This study reports on 77,719 women who gave birth over a 12 month period, of whom 49,281 (63.4%) received a COVID-19 vaccine, 54,887 (70.6%) received an influenza vaccination and 63,594 (81.8%) received a pertussis vaccine by the time of delivery. Pregnant women aged >30 years (aOR 1.31 CI 1.27, 1.36), who had >=8 antenatal visits (aOR 1.08 CI 1.04, 1.12), and those who received influenza vaccine (aOR 1.23 CI 1.19, 1.28) were more likely to have received a COVID-19 vaccine. Those who smoked (aOR 0.7 CI 0.66, 0.74), were First Nations (aOR 0.83 CI 0.74, 0.93) and those who gave birth in public hospitals (aOR 0.65 CI 0.63, 0.68) were less likely to receive COVID-19 vaccine in the first 12 months of the rollout. Conclusion: Maternal age, smoking, parity and Indigenous status were factors associated with delayed and sustained lower coverage, even in a population with background maternal influenza and pertussis coverage of 70.6% and 81.8%, respectively.

## 1. Background

The World Health Organization (WHO) declared the novel coronavirus (SARS-CoV-2) a worldwide pandemic on 11 March 2020. By this time, it had spread rapidly to almost every country, with the first known case in Australia arriving in Melbourne on the 25 January 2020 [1]. The initial impact of the pandemic in Australia was markedly different from many other countries due to border closures and restrictive public health policies. However, like many countries around the world, by the end of 2021 SARS-CoV-2 transmission had become widespread.

Vaccine development during the COVID-19 pandemic was swift and utilised novel technologies, resulting in several vaccines using different vaccine platforms [2]. In Australia, the first vaccines available included BNT162b2 manufactured by BioNTech with Pfizer, mRNA-1273 manufactured by Moderna, AZD1222—initially referred to as ChAdOx1-nCoV-19, now Vaxzevria—manufactured by Astra Zeneca, and later NVX-CoV2373 manufactured by Novavax. Notably, all the initial clinical vaccine trials excluded pregnant women [3,4].

As vaccine development progressed, many reports emerged during the first year of the pandemic suggesting that pregnant women were at an increased risk of severe disease [5,6,7]. It soon became evident that pregnant women were at an increased risk of hospitalisation, admission to the intensive care unit, mechanical ventilation, preterm birth and death. However, in the absence of clinical trial data, pregnant women in Australia were not routinely recommended vaccination during the early stages of the vaccine rollout, which began in Australia on 22 February 2021.

On 9 June 2021, the Australian Technical Advisory Group on Immunisation (ATAGI) and the Royal Australian and New Zealand College of Obstetricians and Gynaecologists (RANZCOG) released a joint statement acknowledging the increased risk of severe disease and routinely recommended vaccination in pregnancy [8]. From 18 July 2021, pregnant women were able to access mRNA vaccines in Australia. 

In Australia, two other maternal vaccines are routinely recommended in every pregnancy: inactivated Influenza Virus (IIV), diphtheria, tetanus and acellular pertussis (dTpa). IIV is recommended with each pregnancy at any gestational age, whereas dTpa vaccine is recommended in each pregnancy from 20 weeks gestation [9]. 

The aim of this study was to track the uptake of a new maternal vaccine (COVID-19) over the first 12 months from the time of national recommendation across a population with high coverage of routine maternal vaccinations (influenza and pertussis) to determine the predictive factors associated with early adoption. Furthermore, we sought to compare the coverage of COVID-19 vaccines with the coverage of influenza and pertussis vaccines over the same time period. 

## 2. Methods

The study was a population-based, cohort study of data on births from 1 July 2021 to 30 June 2022 (the first 12 months of the COVID-19 vaccine rollout that included pregnant women). Data were collected using the Victorian Perinatal Data Collection (VPDC).

The VPDC is a mandated data collection system under the Public Health and Wellbeing Act 2008, whereby all public and private hospitals and private-practising midwives provide data to the VPDC on all births in Victoria. Over 160 data items are collected in compliance with the National Perinatal Minimum Data Set. The VPDC is held by the Consultative Council on Obstetric and Paediatric Mortality and Morbidity. The purpose of the VPDC is to provide population-wide data for the improvement of maternal and infant health outcomes. The data in the VPDC is required to be reported within 30 days of the birth event. This requirement exists for all births, including hospital and at-home births [10].

Four items related to COVID-19 vaccination status during pregnancy were added to the VPDC for all births from 1 July 2021: whether the woman had ever received a COVID-19 vaccine; whether she had received one or more COVID-19 vaccines during pregnancy; the gestation of the first dose received during pregnancy; and the gestation of the second dose received during pregnancy. Gestational age of first or second dose were grouped into trimesters (trimester 1 = 1–12 weeks, trimester 2 = 13–26 weeks, and trimester 3 >= 27 weeks). 

Australian-born status was determined based on the maternal country of birth and was defined as either Australian-born or not Australian-born. Maternal age (younger than 20, 20–29, 30–39, 40 or older), gestational age (before 37 weeks, at 37 or more weeks), parity (first birth, second birth, third or subsequent birth) and the number of antenatal visits were categorised. The number of antenatal visits was categorised as <8 or >=8. This cut-off was derived from the WHO guidelines indicating that women should have a minimum of eight contacts/visits during pregnancy [11]. Australian guidelines suggest ten appointments for primiparous women and seven for multiparous women, although this varies according to the complexity of the pregnancy [12].

A variable was generated to enable a comparison of women who received a vaccine during pregnancy in the first 6 months of availability (“early adopters”) with those who received it after the first 6 months of availability (“late adopters”) and those who had not received a COVID-19 vaccine by the time of birth. All women who gave birth between July and December 2021 were included in the first category, along with those who gave birth between January and June 2022, but had received at least one COVID-19 vaccination in 2021. All others who received a vaccine during pregnancy but whose first dose was in 2022 were allocated to the second group. Women with missing data and those who with only COVID-19 vaccinations before pregnancy were allocated to the ‘other’ category.

### Analysis

Each birth is a separate record in the VPDC, meaning that women appear twice when they have multiple births. We excluded cases with birth order >1 to include women only once in the maternal analyses. Those who declined to answer were considered as “missing”. We used descriptive statistics to analyse the relationship between maternal and maternity care characteristics, receiving at least one COVID-19 vaccination before birth, and the relationship between these characteristics and early adoption of the vaccine. To explore whether the demographic factors associated with delayed and low uptake of COVID-19 vaccines were similar to an existing vaccine or whether this was specific to COVID-19 vaccines, we examined the factors associated with influenza vaccination during pregnancy. We chose to compare it to influenza as historically coverage in pregnancy has been lower than that in pertussis. Proportions were compared using the Chi-square test, and *p*-values < 0.05 were considered statistically significant.

Univariable and multivariable logistic regressions were used to describe the odds ratio of receiving at least one COVID-19 vaccination before birth after adjusting for several maternal and maternity care characteristics. Odds ratios, adjusted odds ratios and 95% confidence intervals were reported. Covariates included in the multivariable analysis were maternal age, whether the woman was born in Australia, Aboriginal status, whether the birth was in a public or private hospital, parity, maternal smoking during pregnancy, number of antenatal visits (<8 versus 8 or more) and whether the woman received influenza vaccine during the pregnancy. Characteristics known to be associated with the uptake of other vaccines during pregnancy were included in the multivariable model. Where collinearity was found, one of the collinear variables was selected for inclusion in the model. Pertussis and influenza vaccination could not both be included due to collinearity, so influenza was selected given historically lower coverage rates, and it is a vaccine directed against a respiratory virus for both maternal and infant benefits.

This project was approved by the Monash University Human Research Ethics Committee (project ID 30927).

## 3. Results

Between 1 July 2021 and 30 June 2022, 77,719 women gave birth to 78,822 babies in Victoria. A total of 49,281 (63.4%) received a COVID-19 vaccine at the time of delivery, 26,099 (33.6%) were not vaccinated and 1828 (2.4%) declined to answer. Vaccination status was unknown for a small fraction of women (excluding those who declined to answer) with only 511 (0.6%) not known. In total, 46,281 women (59.3%) had at least one COVID-19 vaccination during pregnancy, and 2731 were vaccinated before pregnancy only. The overall coverage for influenza vaccination for the same period was 70.6% (54,887/77,719), and for pertussis, it was 81.8% (63,594/77,719). 

Table 1 describes the total number of patients who received at least one COVID-19 vaccine according to the maternal characteristics and aspects of antenatal care. Pregnant women who received antenatal care and gave birth in the private system, those who had >=8 antenatal visits, non-smokers, non-First Nations women, and women over 30 years of age were more likely to be vaccinated (Table 1). Pregnant women who received IIV or dTpa were also more likely to receive a COVID-19 vaccine. Also notable was the decline in coverage according to the level of English proficiency (Table 1). 

**Figure 1:**
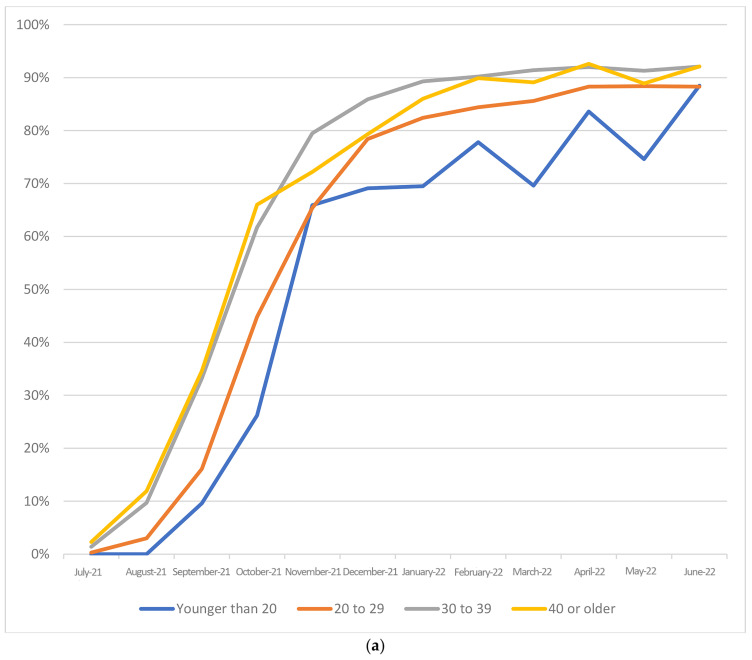

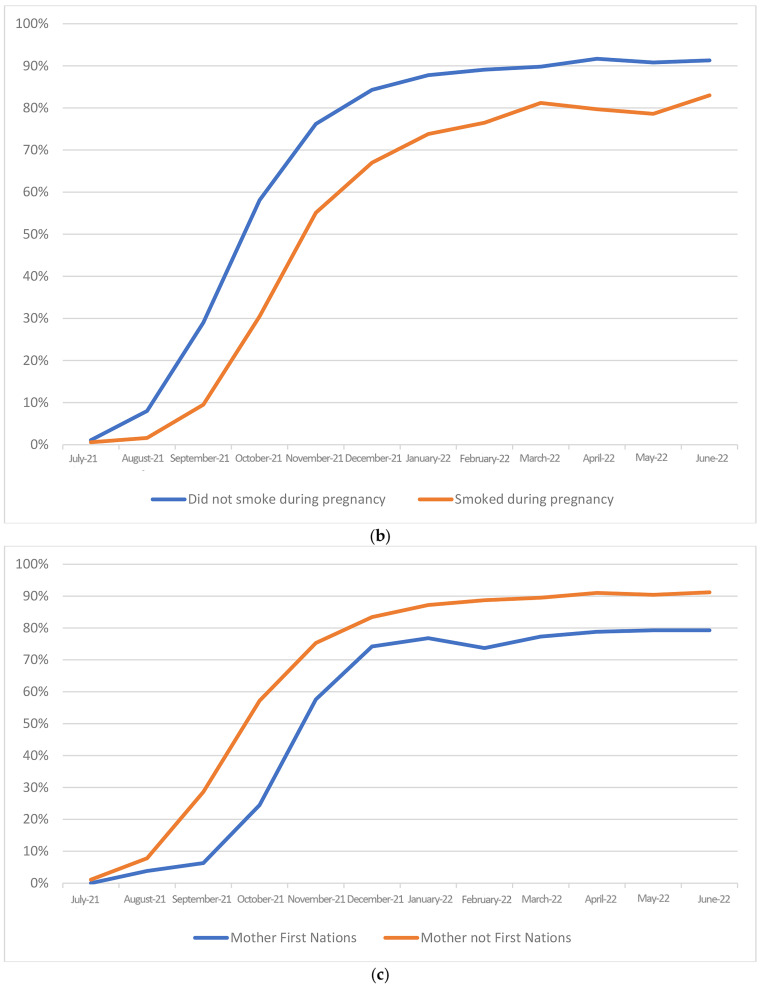

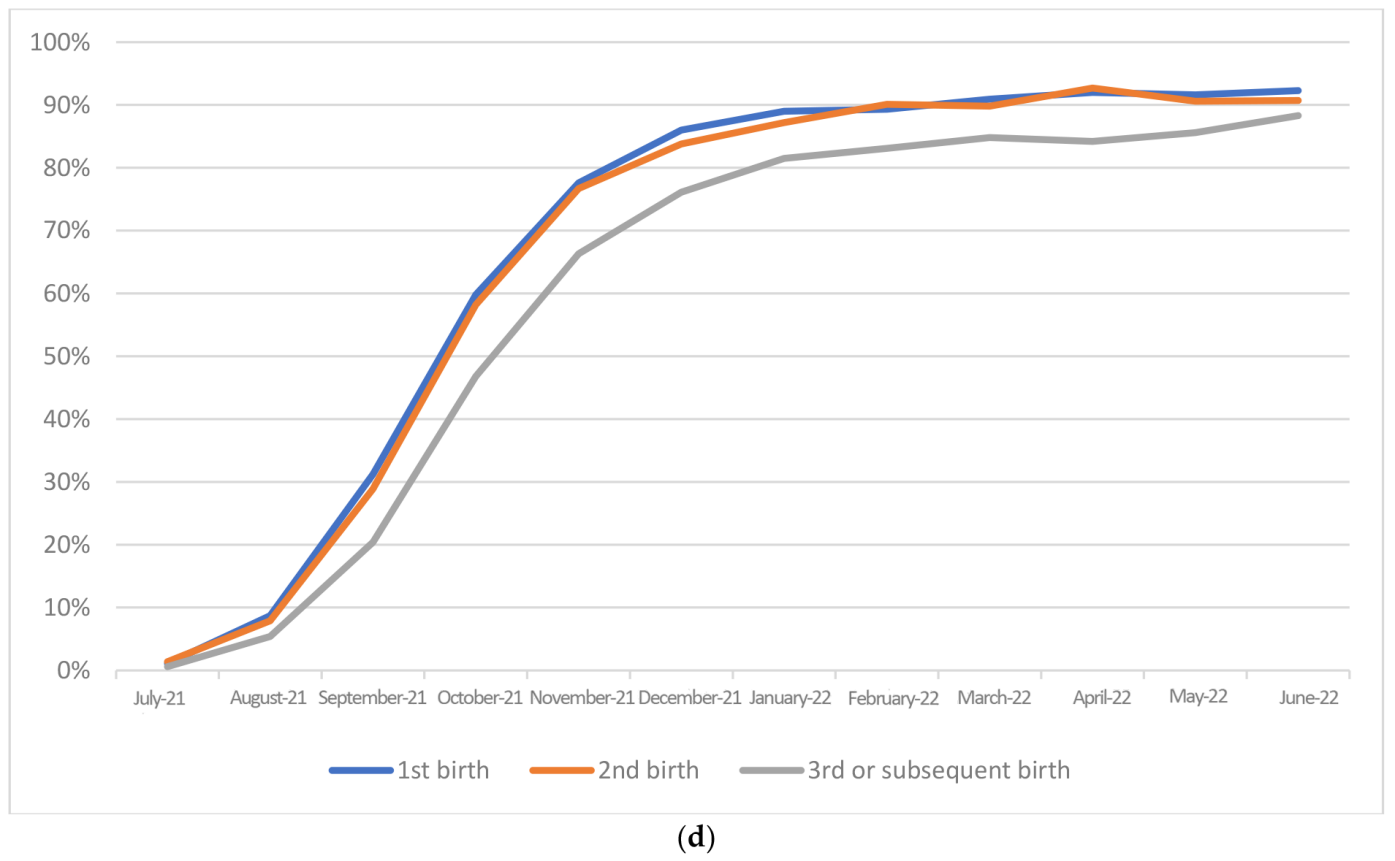
(**a**) Percentage of women who received at least 1 COVID-19 vaccination before birth (including before pregnancy) based on maternal age group and the month baby was born. (**b**) Percentage of women who received at least 1 COVID-19 vaccination before birth (including before pregnancy) based on smoking status and the month baby was born. (**c**) Percentage of women who received at least 1 COVID-19 vaccination based on maternal First Nations status and the month baby was born. (**d**) Percentage of women who received at least 1 COVID-19 vaccination before the birth (including before pregnancy) based on parity and the month baby was born.

Table 2 compares the characteristics of women who accepted vaccination in the first 6 months of availability with those who were vaccinated in 2022. ‘Late adopters’ (those vaccinated in 2022) were more likely to have been born outside Australia, to have a first baby, aged 40 or older, to have started antenatal care after 27 weeks gestation, had fewer than eight antenatal visits and to be First Nations compared to those who were vaccinated in the first 6 months of vaccine availability. They were also less likely to speak English ‘very well’ (if born outside Australia), and to have been vaccinated against influenza (all *p* < 0.001). Figure 1a–d demonstrate the uptake over time for the demographic factors significantly associated with low and delayed uptake (maternal age, parity, First Nations status and smoking).

Maternal age, smoking, parity and First Nations status were factors associated with delayed and sustained lower coverage (Figure 1a–d). 

We compared this uptake of COVID-19 vaccines with the influenza vaccine to examine if there were similar trends according to maternal factors (maternal age, smoking, parity and First Nations status). Women younger than 20 years, First Nations women, women pregnant with their third or subsequent child and smokers were also more likely to have low uptake of influenza.

## 4. Discussion

The key findings in this study that analyses the uptake of COVID-19 vaccines in pregnant women over the first 12 months of the rollout are as follows: 1. The overall uptake was significantly lower compared to other recommended vaccines during pregnancy (IIV and dTPa). 2. Uptake was slower in younger age groups, those who smoked, First Nations populations and women who were pregnant with their third or subsequent child. 3. Uptake was higher in women aged >30, who received influenza vaccine, who had =>8 antenatal visits, who were pregnant with their first baby or who delivered in the private system 4. The overall coverage of influenza was lower in the same groups, who were also slower to take up the COVID-19 vaccine. 

The background maternal vaccine coverage of influenza and pertussis in the setting where this study took place is relatively high compared to the remainder of Australia (81.8% and 84.3% in 2020, respectively) [13]. This suggests high levels of vaccine acceptance among the pregnant population, noting that these vaccines have been recommended in pregnancy in Australia since 2000 for influenza and since 2015 for pertussis. In this context, uptake following the introduction of a new maternal vaccine is notable in achieving an overall coverage of only 63%, even in the setting of vaccine mandates, without exemptions granted for pregnancy [14]. It is likely that concerns about safety may have contributed to the lower overall coverage compared to established vaccines in pregnancy, such as influenza and pertussis, although this was not formally explored in our study. In other studies, factors identified with low uptake of COVID-19 vaccines have included concerns about safety, along with low health literacy and education level [15,16], with concerns about safety being the most commonly cited factor contributing to hesitancy, even among diverse populations [15,16].

Our results demonstrate that after approximately six months of the vaccine being available, uptake plateaued and remained lower in groups known to be at a higher risk of severe disease (e.g., smokers and First Nations people). These findings have also been reported in non-pregnant populations with these risk factors [17]. In January 2022, 92% of non-Aboriginal people in Australia were fully vaccinated compared to 74% of Aboriginal people [18]. Qualitative research among non-pregnant Aboriginal populations cited reasons for low uptake. The reasons cited included distrust, fear of vaccine side effects and a preference for vaccination programmes targeting Aboriginal people to be led and delivered via Aboriginal Health Services, as Aboriginal Health Services were seen as a trusted source of information [19]. Similar themes of distrust, concern about lack of research and fear of side effects have been reported among qualitative interviews of tobacco smokers [17]. We have also previously reported a lower uptake of influenza and pertussis vaccines among pregnant people in their second or third pregnancy compared to their first pregnancy, suggesting that this finding is not specific to COVID-19 vaccines [20]. 

So, how do we interpret these ‘factors’ associated with either slow or low uptake of maternal vaccines and how do we use this information to inform public health policy? For the identification of groups with delayed and sustained low coverage in the antenatal setting, we need to consider targeted public health messages and information working in partnership. For vaccines recommended during pregnancy, this may include delivery via Aboriginal Health Services rather than the antenatal care provider if they are not the same. For smokers, the messages may need to focus on strategies to maximise health rather than just focusing on tobacco consumption, and given the differences found between first and subsequent pregnancies in uptake, messages may need to be tailored to explain the importance of repeat vaccination in each pregnancy. The strength of healthcare provider recommendations and motivation from peers [21,22,23] cannot be underestimated and needs to focus on these groups. This may overcome any underlying hesitation. Furthermore, health systems must address equitable access to maternal vaccines, particularly at the point of antenatal care, as studies consistently demonstrate this to increase uptake and coverage [24,25]. 

What does this mean for future maternal vaccines such as respiratory syncytial virus (RSV) and group B streptococcus (GBS)? These two new maternal vaccines have either completed phase 3 clinical trials or are about to start, with one maternal RSV vaccine recently licensed and available for use in some countries [26,27]. This may mean that they will soon be registered and added to maternal vaccine schedules. Although this will be outside of a pandemic, the lessons learned from the introduction of a new maternal vaccine (COVID-19) should be considered. Early targeted information to groups that show lower uptake of existing vaccines (influenza) and who demonstrated similar delays in receiving new vaccines (COVID-19), bolstering predictors of uptake such as healthcare providers and peer recommendations, and improving access at the point of antenatal care will be key to the success of any future maternal immunisation added to a national schedule [25].

Our study has strengths and limitations. The main strength is that the perinatal data collection system used in Victoria, Australia, introduced mandatory reporting of COVID-19 vaccination status to its perinatal data collection from 1 July 2021. This allowed us to observe and report on the trends over the first 12 months of the rollout correlating with when pregnant women were recommended and able to access free COVID-19 vaccines. The other strength of this dataset is that it includes every pregnancy, not restricted to live births, across the private and public healthcare settings. This allowed for the timely analysis of COVID-19 vaccine coverage based on demographics. The limitation of this study is that it is observational in nature; therefore, not all possible confounders contributing to uptake can be accounted for, such as occupation, educational level, comorbidities and socioeconomic status. As an example, the number of antenatal visits was categorised into <8 or >=8 as a surrogate for the opportunity of vaccination and engagement in healthcare systems. However, a pregnant woman may have had more visits because she was at a ‘high-risk’ pregnancy, and therefore may have had a healthcare provider recommendation or other risk factors for severe COVID-19 disease leading to earlier vaccination, or she may have had fewer visits because she gave birth preterm. 

The considerations for maternal immunisation programmes based on factors associated with low uptake in this study are outlined below:

- Public health messages need to consider investing greater effort in younger women who may perceive themselves as “healthy”;
- Providers need to understand that women in their third or more pregnancy may need more information about “why” they are still recommended to receive vaccines when they may have received vaccines in prior pregnancies;
- Women with risk factors for respiratory illness such as smokers may need targeted messaging-, e.g., “if you are unable to stop smoking during pregnancy then there are measures you can consider to improve your health and that of your baby”;
- English proficiency, rather than country of birth, influences maternal vaccine uptake.

## 5. Conclusions

Maternal age, smoking, parity and Indigenous status were factors associated with delayed and sustained lower coverage. Identifying these groups will help in directing public health messaging not just for existing maternal vaccines but new maternal vaccines on the horizon.

## Figures and Tables

**Table 1:** At least one COVID-19 vaccination before birth, including pre-pregnancy, for all women who gave birth in Victoria during July 2021–June 2022.

	YES (N = 42,281)		NO (N = 26,099)						
	n	%	n	%	*p*-Value	OR	95%CI	aOR	95%CI
Hospital type									
Public	36,119	62.7	21,478	37.3	<0.001	0.57	(0.55, 0.59)	0.65	(0.63, 0.68)
Private	13,018	74.8	4390	25.2					
Mother born in Australia									
Yes	30,576	65.1	16,405	34.9	0.12	0.98	(0.95, 1.01)	1.03	(1.00, 1.07)
No	18,285	65.6	9571	34.4					
Parity									
First birth	22,566	67.4	10,913	32.6	<0.001	1.42	(1.37, 1.48)	1.37	(1.31, 1.43)
2nd birth	18,005	66.2	9198	33.8		1.35	(1.29, 1.41)	1.25	(1.20, 1.31)
3rd or more	8710	59.3	5988	40.7		ref			
Gestation at first antenatal visit									
4–13 weeks	39,845	66.0	20,564	34.0	<0.001			ne	
14–27 weeks	8394	63.7	4776	36.3					
>27 weeks	733	60.0	488	40.0					
Number of antenatal visits									
<8	10,906	61.8	6746	38.2	<0.001				
>=8	37,975	66.4	19,229	33.6		1.22	(1.18, 1.26)	1.08	(1.04, 1.12)
Maternal smoking at any time during this pregnancy									
No	44,156	66.1	22,660	33.9	<0.001				
Yes	2835	52.2	2591	47.8		0.56	(0.53, 0.59)	0.7	(0.66, 0.74)
Maternal First Nations status									
Yes	641	52.2	587	47.8	0.001	0.57	(0.51, 0.63)	0.83	(0.74, 0.93)
No	48,544	65.6	25,440	34.4					
Received influenza vaccine during this pregnancy									
Yes	35,969	67.2	17,567	32.8	0.001	1.33	(1.28, 1.37)	1.23	(1.19, 1.28)
No	12,749	60.7	8241	39.3					
Received pertussis vaccine during this pregnancy									
Yes	41,704	67.3	20,309	32.7	0.001	1.59	(1.53, 1.65)	ne	
No	7131	56.5	5495	43.5					
Maternal age (years)									
Younger than 20	344	52.0	317	48.0	<0.001	0.72	(0.62, 0.84)	0.81	(0.69, 0.95)
20–29	13,161	60.1	8763	40.0		ref			
30–39	33,100	67.8	15,701	32.2		1.4	(1.36, 1.45)	1.31	(1.27, 1.36)
40 or older	2675	67.1	1309	32.9		1.37	(1.28, 1.47)	1.29	(1.20, 1.39)
Discipline of main antenatal care provider									
Obstetrician	30,141	67.5	14,502	32.5	0.001			ne	
Midwife	15,524	62.8	9199	37.2					
General practitioner	3287	60.9	2114	39.1					
None	57	31.1	126	68.9					
Other	193	68.0	91	32.0					
Maternal proficiency in spoken English *									
Very well	15,074	67.7	7184	32.3	<0.001				
Well	4988	66.3	2530	33.7					
Not very well	1435	62.6	857	37.4					
Not at all	454	61.8	281	38.2					

* excludes women born in Australia, ref = reference, ne = not entered (variable excluded due to collinearity).

**Table 2:** Characteristics of early adopters of COVID-19 vaccination, late adopters, unvaccinated and unclassified women, for all women who gave birth in Victoria during July 2021–June 2022.

	Early Adopter		Late Adopter		Unvaccinated		Unclassifiable			
	n	%	n	%	n	%	n	%	*p*-Value (All Groups)	(Early vs. Late Adopters
Hospital type										
Public	32,527	75.4	2051	75.5	21,478	82.3	3208.0	56.0	<0.001	0.796
Private	10,503	24.3	662	24.4	4389	16.8	2456.0	42.9		
Other	108	0.3	5	0.2	231	0.9	61.0	1.1		
Mother born in Australia										
Yes	26,810	62.1	1613	59.4	16,405	62.9	3633.0	63.5	<0.001	0.002
No	16,049	37.2	1074	39.5	9570	36.7	1986.0	34.7		
Not reported	319	0.7	31	1.1	123	0.5	106.0	1.9		
Parity										
First birth	19,691	45.6	1288	47.4	10,913	41.8	2542.0	44.4	<0.001	0.185
2nd birth	15,804	36.6	968	35.6	9197	35.2	2039.0	35.6		
3rd or more	7683	17.8	462	17.0	5988	22.9	1144.0	20.0		
Gestation at first antenatal visit										
4–13 weeks	34,880	80.8	2138	78.7	20,563	78.8	4693.0	82.0	<0.001	<0.001
14–27 weeks	7466	17.3	481	17.7	4776	18.3	750.0	13.1		
>27 weeks	577	1.3	76	2.8	488	1.9	160.0	2.8		
Not reported	255	0.6	23	0.9	271	1.0	122.0	2.1		
Number of antenatal visits										
<8	9549	22.1	678	24.9	6746	25.9	1383.0	24.2	<0.001	0.002
>=8	33,291	77.1	2022	74.4	19,232	73.7	4268.0	74.6		
Not reported	338	0.8	18	0.7	120	0.5	74.0	1.3		
Maternal smoking at any time during this pregnancy										
No	38,714	89.7	2417	88.9	22,659	86.8	5076.0	88.7	<0.001	0.001
Yes	2604	6.0	144	5.3	2630	10.1	339.0	5.9		
Not reported	1860	4.3	157	5.8	809	3.1	310.0	5.4		
Maternal First Nations status										
Yes	568	1.3	46	1.7	598	2.3	88.0	1.5	<0.001	0.243
No	42,540	98.5	2667	98.1	25,439	97.5	5609.0	98.0		
Not reported	70	0.2	5	0.2	61	0.2	28.0	0.5		
Received influenza vaccine during this pregnancy										
Yes	31,590	73.2	1947	71.6	17,566	67.3	3784.0	66.1	0.001	0.192
No	11,122	25.8	733	27.0	8241	31.6	1666.0	29.1		
Not reported	466	1.1	38	1.4	291	1.1	275.0	4.8		
Received pertussis vaccine during this pregnancy										
Yes	37,031	85.8	2345	86.3	20,309	77.8	3909.0	68.3	0.001	0.049
No	5795	13.4	339	12.5	5494	21.1	1531.0	26.7		
Not reported	352	0.8	34	1.3	291	1.1	282.0	4.9		
Maternal age (years)										
Younger than 20	307	0.7	19	0.7	317	1.2	45.0	0.8	<0.001	<0.001
20–29	11,787	27.3	699	25.7	8762	33.6	1413.0	24.7		
30–39	28,891	66.9	1819	66.9	15,701	60.2	3818.0	66.7		
40 or older	2193	5.1	180	6.6	1309	5.0	447.0	7.8		
Not reported	-	0.0	<5	0.0	9	0.0	<5	0.0		
Discipline of main antenatal care provider										
Obstetrician	25,781	59.7	1678	61.7	14,501	55.6	4173.0	72.9	<0.001	0.043
Midwife	14,157	32.8	855	31.5	9199	35.3	1105.0	19.3		
General practitioner	2976	6.9	165	6.1	2114	8.1	314.0	5.5		
None	41	0.1	7	0.3	126	0.5	60.0	1.1		
Other	151	0.4	10	0.4	91	0.4	44.0	0.8		
Not reported	72	0.2	3	0.1	67	0.3	29.0	0.5		
Maternal proficiency in spoken English *										
Very well	13,167	30.5	940	34.6	7184	27.5	1535.0	26.8	<0.001	<0.001
Well	4299	10.0	277	10.2	2529	9.7	617.0	10.8		
Not very well	1266	2.9	84	3.1	857	3.3	165.0	2.9		
Not at all	408	0.9	31	1.1	281	1.1	64.0	1.1		
Not reported	1063	2.5	72	2.7	381	1.5	191.0	3.3		

* excludes women born in Australia.

## Data Availability

Data is unable to be shared due to restrictions on its use.
